# Disaster Resilience Reduces Radiation-Related Anxiety Among Affected People 10 Years After the Fukushima Daiichi Nuclear Power Plant Accident

**DOI:** 10.3389/fpubh.2022.839442

**Published:** 2022-07-14

**Authors:** Tomoyuki Kobayashi, Masaharu Maeda, Chihiro Nakayama, Yui Takebayashi, Hideki Sato, Noriko Setou, Maho Momoi, Naoko Horikoshi, Seiji Yasumura, Hitoshi Ohto

**Affiliations:** Fukushima Medical University School of Medicine, Fukushima, Japan

**Keywords:** resilience, psychological distress, radiation risk anxiety, discrimination, Fukushima nuclear disaster

## Abstract

This study examined whether disaster resilience affects the recovery of mental health states and mitigates psychosocial anxiety 10 years later the Fukushima Daiichi nuclear power plant accident. The survey was conducted in Fukushima's evacuation-directed and non-evacuation-directed areas in January 2020. The 695 participants responded to a questionnaire including items on radiation-related anxiety regarding the Fukushima Daiichi accident, an action-oriented approach as a resilience factor, psychological distress, and demographic information. The structural equation modeling showed that the action-oriented approach also eased radiation-related anxiety by mediating with improving mental health states. Moreover, a multi-group model analysis was conducted for evacuation-directed and non-directed areas. In the evacuation-directed area, we found stronger associations among resilience, mental health states, and radiation-related anxiety, and a direct effect of resilience factors on radiation risk anxiety. These findings emphasize the importance of resilience in post-disaster contexts, at least for a decade, where mental health deteriorates and various psychosocial issues become more complex.

## Introduction

A severe earthquake and tsunami caused the Fukushima Daiichi nuclear power plant accident in March 2011. Fukushima is located in the northeast region of Japan and has the plant in the area facing the Pacific Ocean. Three reactors were melded down and a large amount of radioactive substances were released into environment for 5 days, which was evaluated as the most severe level by the International Nuclear and Radiological Event Scale. The Japanese government requested mandatory or voluntary evacuation of areas more than 20 km from the plant. In Fukushima, over 154,000 residents were required to evacuate long-term.

This traumatic event has raised several psychosocial problems in the long term. It includes concerns about getting thyroid cancer that was observed after the Chornobyl accident. Although international organizations reported no discernible physical risks of the radiation exposure in Fukushima (1), the residents' anxiety levels have affected several aspects of their lives, such as food safety, the intention to return to one's home after evacuation, and collision with family members by differences in radiation risk perception (2). The health concerns also included control of chronic diseases was extremely difficult during the evacuation because of reduced access to medical care due to poor accessibility to their doctors and their hospitals, reduced physical activity, and changes in diet. In addition, this nuclear accident caused social issues such as discrimination. Evacuees have experienced shunning from their neighbors and difficulties finding employment because they were prejudiced by being perceived as if they are radioactive or as if they made a fortune from compensation (3). These problems have produced mental health problems such as depression symptoms, post-traumatic stress disorder (PTSD), alcohol misuse, and suicide (4, 5).

Despite such serious traumatic events, some people could be resilient, that is, they can trace to recover their mental health and adapt to life after the event. Orui et al. (6) surveyed Fukushima residents 7 years after the accident and identified the pattern of the residents' resilience process. More than 80% of the participants with deteriorated mental health states following the accident reported recovery soon after. Resilience is defined as the process of, capacity for, or outcome of successful adaptation despite challenging or threatening circumstances (7). Factors that contribute to successful resilience involve not only personal traits but also behaviors, thoughts, and actions that anyone can learn and develop (8).

Takebayashi et al. (9) interviewed the residents affected by the accident and revealed that an action-oriented approach as a factor of their resilience capacity was associated with a decline in their psychological distress following the accident. This action-oriented approach represents an acceptance of discomforts in life after the disaster, engaging in hobbies and new activities, and participating in social networks. Previous studies have focused on each aspect of the action-oriented approach as a factor of resilience development in various contexts: Physical and psychological avoidance that traumatic experiences bring affected people can lead to clinically serious distress and dysfunction in the form of depression and PTSD (10). However, people who accept the context are more likely to control the emotions by reducing avoidant attitudes toward their traumatic experiences (11). For example, a nationwide survey of individuals shortly after the terrorist attacks in the USA found reduced levels of posttraumatic stress symptoms in those who accepted the situation (12). Additionally, leisure activities and social participation could reduce mental health problems such as depression (13). In particular, in social participation, both receiving and providing social support are important for improving mental health after a traumatic experience (14, 15).

Resilience is key to individual recovery in post-disaster life. However, little is known on how resilience relates to reducing psychosocial problems specific to a particular disaster, extending from the recovery of mental health states. The current study reconfirmed that resilience accelerates the mental health recovery of the affected people in the Fukushima Daiichi accident, and examined whether resilience also contributes to the reduction of psychosocial problems specific to this nuclear accident.

Furthermore, causal relationships between mental health and other psychosocial problems (such as radiation and discrimination anxiety) in the context of the Fukushima Daiichi accident have been controversial. Takebayashi et al. (2) reviewed research on radiation-risk anxiety related to the accident from 2011 to 2017 and showed that such anxiety increased severe psychological distress. Living with the concern of the effects of radiation on oneself and one's family is stressful and is likely to lead to the development of some psychological disorder. On the contrary, researchers often find that radiation risk anxiety and perception about risk are based on mental health states related to or unrelated to the context or an event (16). For example, Sobkow et al. (17) show that participants in stress conditions that introduce a risk-irrelated stress task were likely to perceive risk in various risky situations as higher than the control condition. Although few studies have examined the latter pattern in the context of the Fukushima Daiichi accident, Suzuki et al. (18), in a longitudinal survey, report that people with strong traumatic reactions in 2011 were likely to perceive a higher risk of the delayed and genetic effects of radiation until at least 2 years later.

Therefore, the current study has two purposes: one is to clarify the causality among resilience factors, psychological distress, and psychosocial factors such as radiation risk and discrimination; and the other is to examine some differences in the causality between evacuation-directed and non-directed areas in Fukushima prefecture.

## Methods

### Participants

**Figure 1:**
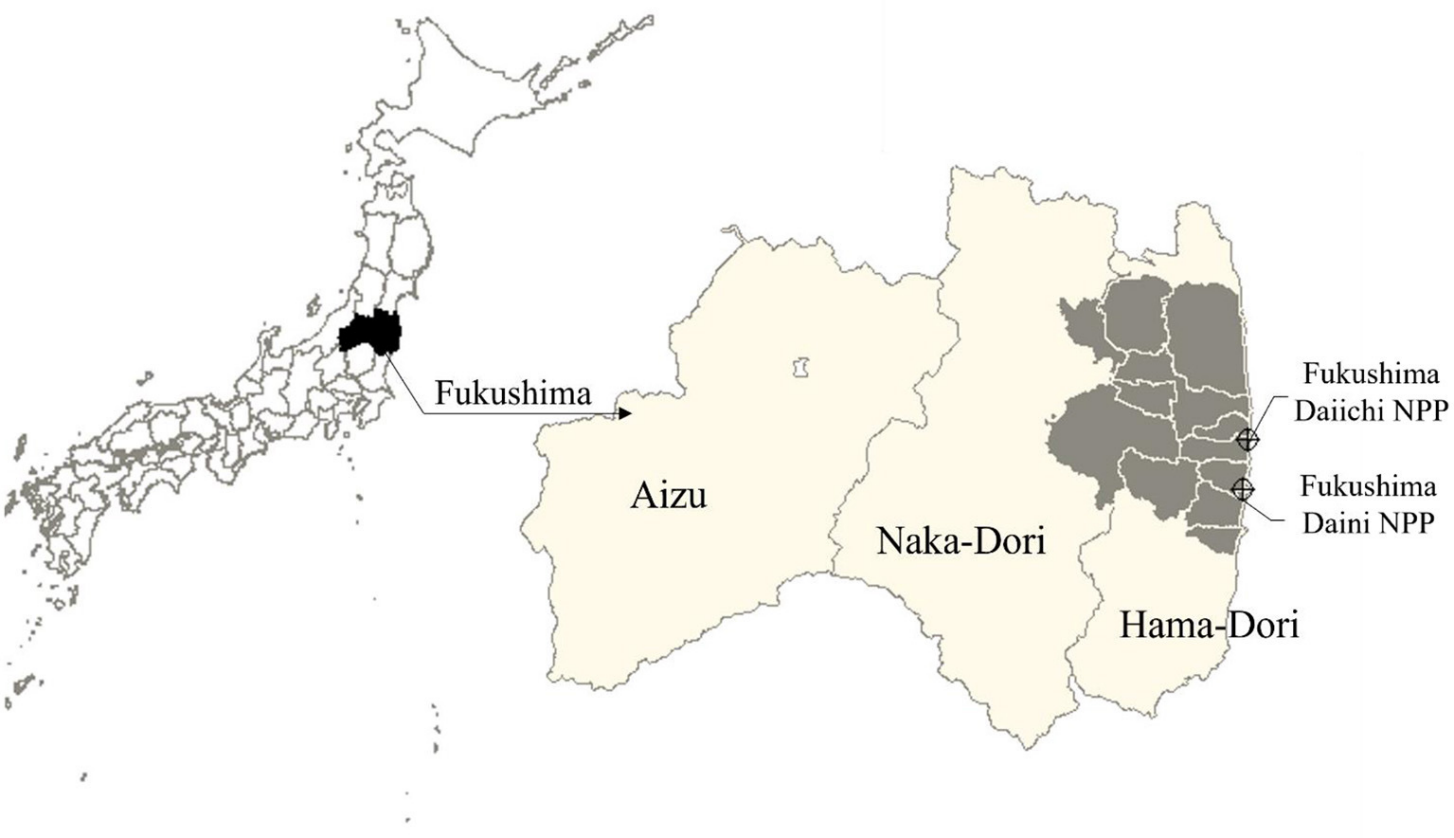
Map of Fukushima Prefecture. These maps have the north on top. The map of left shows Japan and the map of right shows Fukushima Prefecture. In the Fukushima's map, the gray areas are municipalities with evacuation-directed areas.

This survey was conducted for 1,600 Fukushima prefecture residents in January 2020. A two-step stratified random sampling was applied. Fukushima prefecture comprises three regions separated vertically by two mountain ranges: Aizu, Naka-Dori, and Hama-Dori (Figure 1). The Hama-Dori area faces the Pacific Ocean and has two nuclear power plants, including the Fukushima Daiichi nuclear power plant. At the accident, the evacuation directions from the Japanese government have mainly designated for some towns in Hama-Dori. Because we distinguish those who have experienced forced condemnation in the analysis, 400 residents were selected from each of the four areas: Aizu, Naka-Dori, the evacuation-directed area in Hama-Dori, and the non-evacuation-directed area in Hama-Dori. The residents received a postal envelope with a questionnaire. Participants gave informed consent in writing on the questionnaire and expressed their agreement by submitting the questionnaire. With regard to this survey, an association among a continuance of radiation risk anxiety, life style, and media utilization has been reported previously (19).

### Survey Questions

The questionnaire included items on radiation-related anxiety regarding the Fukushima disaster, resilience, psychological distress, and demographic information.

Radiation-related anxiety was measured to assess psychosocial problems related to nuclear disasters. It was assessed by using the seven items of Umeda et al. (20). These items asked about health concerns following the radiation exposure and other problems due to the Fukushima disaster: “I am afraid that in the future I will get a serious disease due to radiation;” “Every time I get sick, I worry that it is because of radiation exposure;” “I am afraid that the effects of radiation will be inherited by my children, grandchildren, and other future generations;” “I get very anxious when watching news reports about the nuclear power plant accident;” “I am worried that my children and I will be discriminated against (or treated unfairly) because we live in an area where radiation levels are considered to be high;” “I avoid telling everyone that I am a resident of that area;” and “I have had conflicting opinions with my family about the effects of radiation on human health.” Participants rated them from 1 (totally disagree) to 4 (totally agree).

The resilience was assessed with four items of the action-oriented approach, which is a subscale of the Fukushima Resilience Scale (9). This scale was developed based on interviews on resilience with residents in the evacuation zone in Fukushima. This subscale was extracted by factor analysis and the predictive validity for psychological distress was confirmed (9). Participants rated each item from 1 (disagree) to 5 (agree).

The psychological distress was assessed by the Japanese version (21) of the six-item Kessler Psychological Distress Scale [K6; (22)]. The K6 is typically used to screen for mood or anxiety disorders (21). Responders with five or more points were classified as having psychological distress. Participants rated each item from 0 (none of the time) to 4 (all of the time). Finally, participants were asked their sex, age, and educational background.

### Statistical Design

We conducted an exploratory factor analysis on the seven items of radiation-related anxiety. The number of factors was determined based on a parallel analysis and a minimum average partial correlation. For the exploratory factor analysis, Promax rotation and the maximum likelihood method were used. The cut off point for loadings was 0.5. Moreover, we conducted a confirmatory factor analysis on the four items of resilience.

To investigate the effect of resilience on radiation-related anxiety and psychological distress, we examined a multi-group model of the evacuation-directed and non-directed areas. We compared the fit indices of two models, which have different hypotheses about causal relationships between radiation-related anxiety and psychological distress. The first model is a mediation model in which resilience mitigates psychological distress via radiation-related anxiety. The second model is a mediation model in which resilience mitigates radiation-related anxiety *via* psychological distress. We assumed measurement models that predicted the factors from their observed item scores for resilience and radiation-related anxiety, which were dependent on the results of factor analyses. A chi-square test compared the models. Next, the measurement invariance of the model that was observed to have better fit indices was tested through changes of model fit by gradually adding constraints. We assessed that the invariance hypothesis is not rejected when a loss in the comparative fit index (CFI) <0.01 (23). When invariance is hypothesized in the model, we tested for differences in the values of the path coefficients. We then analyzed the mediation effects in the multi-group model. Although K6 score are often cutoffs based on 5 and 13 scores (21, 22), these models used the total of the six items as the psychological distress variable. These models were also controlled for sex (0 = men, 1 = women), age (0 = under 65 years old, 1 = 65 years old or over), and educational background (0 = junior or senior high school, 1 = vocational college, university, graduate school).

In all structural equation modeling (SEM), we used the means of weighted least squares and variance-adjusted estimation. Model fit was assessed using CFI, standardized root mean square residual (SRMR), and root mean square error of approximation (RMSEA). A model was typically accepted as a good fit when CFI > 0.95, SRMR <0.08, and RMSEA <0.06 (24). The significance level was 5%.

All analyses were performed in R statistical software (R Core Team) (25), using the tools “psych” for the minimum average partial correlation and parallel analysis, and an exploratory factor analysis (26), and “lavaan” for SEM (27). We considered findings significant when *p*s < 0.05.

### Ethics

This study was approved by the Ethics Committee of Fukushima Medical University (approval “general 2019-110”).

## Results

### Demographic Information of Participants

A total of 695 residents participated (response rate: 43.4%). Of these, 138 residents lived in the evacuation-directed area and 557 residents lived in the non-directed area. The participants in the evacuation-directed area consisted of 76 men and 62 women, and their average age was 62.41 (SD = 14.55) years old. The participants in the non-directed area consisted of 260 men and 297 women, and their average age was 58.05 (SD = 15.67) years old. Table 1 shows more detail information.

**Table 1:** Demographic information of participants.

		**All**	**Aizu**	**Naka-Dori**	**Non-evacuation-directed area in Hama-Dori**	**Evacuation-directed area in Hama-Dori**
***n***		**695**	**190**	**195**	**172**	**138**
Sex	Men	336 (48.3%)	102 (53.7%)	88 (45.1%)	70 (40.7%)	76 (55.1%)
	Women	359 (51.7%)	88 (46.3%)	107 (54.9%)	102 (59.3%)	62 (44.9%)
Age	Mean ± SD	58.92 ± 15.55	60.04 ± 14.14	56.25 ± 16.34	57.90 ± 16.32	62.41 ± 14.55
Education background	Junior high school	120 (17.3%)	24 (12.6%)	25 (12.8%)	31 (18.0%)	40 (29.0%)
	Senior high school	324 (46.6%)	83 (43.7%)	89 (45.6%)	93 (54.1%)	59 (42.8%)
	Vocational college	147 (21.2%)	45 (23.7%)	43 (22.1%)	32 (18.6%)	27 (19.6%)
	University, Graduate school	102 (14.7%)	38 (20.0%)	38 (19.5%)	16 (9.3%)	10 (7.2%)
K6	≥13	34 (4.9%)	4 (2.1%)	9 (4.6%)	6 (3.5%)	15 (10.9%)
	5–13	217 (31.2%)	55 (28.9%)	50 (25.6%)	57 (33.1%)	55 (39.9%)
	<5	421 (60.6%)	125 (65.8%)	130 (66.7%)	106 (61.6%)	60 (43.5%)

*K6 scores of ≥5 suggest suspicion of psychological distress and scores of ≥13 suggest suspicion of affective or anxiety disorder such as depression*.

### Factor Analyses of the Anxiety and Resilience Levels Following the Fukushima Disaster

The radiation-related anxiety following the Fukushima disaster (the seven-item set) was suggested to have one to three factors through parallel analysis and minimum-average partial correlation. We conducted an exploratory factor analysis as the two-factor structure can indicate more persuasion (Table 2). The first factor could be interpreted as “radiation risk anxiety” because the items with high loadings indicated anxiety for health risk due to radiation. The second factor could be interpreted as “discrimination anxiety” because items with high loadings indicated anxiety against being discriminated. The variance explained by these two factors was 30.1 and 20.6%, respectively. Resilience (the four-item set) was shown to have a one-factor structure model by the confirmative factor analysis (CFI = 0.995, SRMR = 0.017, RMSEA = 0.030).

**Table 2:** Exploratory factor analysis of the anxiety following the Fukushima disaster.

		**Factor loadings^b^**	
	**Mean ±SD^a^**	**I**	**II**
I am worried about developing a severe disease in the future due to radiation.	2.24 ± 0.85	0.984	
Whenever I get sick, I worry about the effects of radiation exposure.	1.69 ± 0.78	0.710	
I am worried that the effects of radiation will be passed on to my future generations, such as children and grandchildren.	2.24 ± 0.94	0.610	
News about the nuclear accident makes me anxious.	2.77 ± 0.85		
I am worried that my children and I will be discriminated against because we lived in an area where radiation levels are said to be high.	2.31 ± 0.90		0.801
I avoid telling people that I am a resident of the area.	1.89 ± 0.92		0.644
I have had conflicting opinions with my family about the effects of radiation on health.	1.63 ± 0.83		
Factor correlation			0.732

a *Participants rated each items from 1 (totally disagree) to 4 (totally agree)*.

b *Maximum likelihood and Promax rotation. Loadings > 0.5 were shown. Factor I: radiation risk anxiety, Factor II: discrimination anxiety*.

### Multi-Group Analysis for Evacuation-Directed and Non-directed Areas

#### Comparison Between Two Moderation Models

The multi-group analysis was performed on two mediation models in which resilience mitigates psychological distress *via* radiation-related anxiety (CFI = 0.912, SRMR = 0.064, RMSEA = 0.085) and in which resilience mitigates radiation-related anxiety *via* psychological distress (CFI = 0.961, SRMR = 0.045, RMSEA = 0.057). The former did not have good model fit indicators, and the chi-square test showed that the latter model is a better fit as regards data [$\Delta \chi_{\left(1\right)}^{2}$ = 40.62, *p* < 0.001].

#### Measurement Invariance

The test of measurement invariance showed that the loss of CFI did not exceed 0.01 in the model, with the addition of the constraint of factor loadings (CFI = 0.963) from the configural model (CFI = 0.961). However, further adding the constraint of thresholds, the model was suspected to be unidentified because the variance-covariance matrix of the estimated parameters was not positive definite. Thus, we judged that the model could be assumed a weak measurement invariance (CFI = 0.963, SRMR = 0.046, RMSEA = 0.054).

#### Analyses of Mediation Effects in the Multi-Group Model

**Figure 2:**
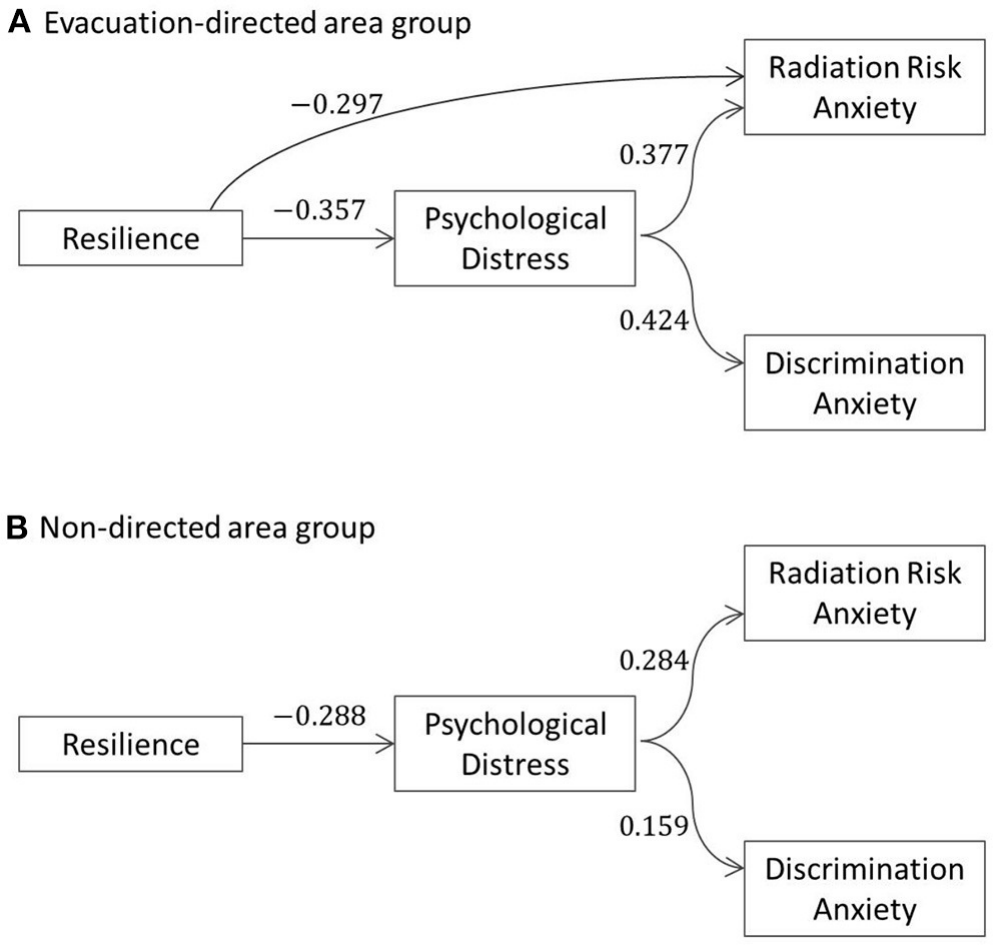
Mediation model from resilience to radiation risk and discrimination anxiety *via* psychological distress. **(A)** Evacuation-directed area group. **(B)** Non–directed area group. Path coefficients are standardized regression coefficients; only statistically significant paths are shown.

Figure 2 shows models in the evacuation-directed area and the non-directed area group. In the evacuation-directed area group, the model showed that the resilience significantly reduced the psychological distress (standardized *b* = −0.357, *SE* = 0.132, *p* = 0.007) and directly affected the radiation risk anxiety (standardized *b* = −0.297, *SE* = 0.147, *p* = 0.043), but did not significantly and directly affect the discrimination anxiety (standardized *b* = 0.014, *SE* = 0.164, *p* = 0.931). The model also showed that the psychological distress significantly affected radiation risk anxiety (standardized *b* = 0.377, *SE* = 0.117, *p* = 0.001) and discrimination anxiety (standardized *b* = 0.424, *SE* = 0.116, *p* < 0.001).

Moreover, the mediation effect was observed on the path between resilience and radiation risk anxiety *via* psychological distress (indirect effect: standardized *b* = −0.136, *SE* = 0.074, *p* = 0.043; total effect: standardized *b* = −0.421, *SE* = 0.140, *p* = 0.013). Whereas, the mediation effect on the path between resilience and discrimination anxiety *via* psychological distress was observed only with an indirect effect (indirect effect: standardized *b* = −0.151, *SE* = 0.077, *p* = 0.050; total effect: standardized *b* = −0.137, *SE* = 0.159, *p* = 0.389).

In the non-directed area group, the model showed that the resilience significantly reduced psychological distress (standardized *b* = −0.288, *SE* = 0.068, *p* < 0.001) and did not significantly and directly affect radiation risk anxiety (standardized b = −0.003, *SE* = 0.067, *p* = 0.965) and discrimination anxiety (standardized *b* = 0.086, *SE* = 0.075, *p* = 0.255). The model also showed that psychological distress significantly affected radiation risk anxiety (standardized *b* = 0.284, *SE* = 0.058, *p* < 0.001) and discrimination anxiety (standardized *b* = 0.159, *SE* = 0.062, *p* = 0.010).

About the mediation effect between resilience and radiation risk anxiety *via* psychological distress, an indirect effect was observed (standardized *b* = −0.082, *SE* = 0.025, *p* = 0.001) but a total effect was not observed (standardized *b* = −0.085, *SE* = 0.064, *p* = 0.186). Additionally, regarding the mediation effect between resilience and discrimination anxiety *via* psychological distress, an indirect effect was observed (standardized *b* = −0.046, *SE* = 0.020, *p* = 0.023) but a total effect was not observed (standardized *b* = 0.040, *SE* = 0.073, *p* = 0.585).

## Discussions

This study examined whether resilience affects the recovery of mental health states and the mitigation of psychosocial anxiety 10 years later the Fukushima Daiichi nuclear power plant accident. Consistent with previous studies (9), the resilience factor (an action-oriented approach) was confirmed to promote the reduction of psychological distress in 2020. Thus, acceptance of the disaster context and engaging in leisure activities and social participation were likely to help recover mental health states a decade later the accident (6, 9). In addition, this study suggests that psychological distress affects two psychosocial problems specific to nuclear accidents: radiation risk anxiety and discrimination anxiety. The resilience factor was found to reduce radiation-related anxiety through reducing psychological distress.

Few resilience studies on the Fukushima Daiichi accident have examined the relationship between resilience factors and psychosocial problems, especially those specific to nuclear disasters. This study found that the action-oriented approach has additional effects beyond the recovery of mental health states. People with deteriorating mental health are likely to hold negative emotions in daily life. Negative emotions tend to hold on to the negative information about risk. Although experts have improved the methods and contents of radiation-related information, it often contains false and conspiracy theories even now. Therefore, people with deteriorating mental health are likely to have radiation-related anxiety because of holding on to these theories (28). When their mental health improves through resilience, they are less likely to attend to negative information about radiation, which may reduce their anxiety.

Moreover, this study analyzed evacuation-directed and non-directed areas separately. The standardized partial regression coefficients were higher for the evacuation-directed area group. This finding suggests that the process paths were more intense in the evacuation-directed area group because they were likely to have frequently faced fears of radiation risk and discrimination during the evacuation. Furthermore, the models show that the action-oriented approach, one of the most significant factors of resilience among the Fukushima evacuees, directly and totally affected radiation risk anxiety in the evacuation-directed area. According to Murakami et al. (29), living in Fukushima decreases risk perception because of the increased exposure to communication about radiation risk. People who take the action-oriented approach can more positively face the chances for communication because they are more likely to join the community. In the evacuation-directed area, there are more chances for communication within the community. However, the current study also suggests that the direct effect of the action-oriented approach is limited as it did not significantly affect discrimination anxiety. A persuasive reason is that people may perceive discrimination as mainly coming from outside the community. Although the action-oriented approach includes action regarding the construction of social relationships, it may reduce anxiety regarding nuisance from the inside, not from the outside. Therefore, even resilient people may not have reduced discrimination anxiety when remaining concerned about discrimination from outside. Discrimination anxiety can lead to poor mental health and reduced quality of life. Future studies should examine relationships between resilience and discrimination anxiety following a disaster.

Furthermore, the current study supports the model that mental health states predict other psychosocial problems related to the nuclear accident, suggesting the different hypotheses of previous studies. The models in previous studies assumed that the presence of various psychosocial problems prompted the deterioration of mental health. The result of the current study may not contradict previous models. Both causal relationships may have existed since the accident. In the early years, radiation risk anxiety may have been strongly influenced by information confusion and groundless rumors that have since been organized. With easy access to accurate information even when anxious, radiation risk anxiety caused by information confusion has diminished. As a result, radiation risk anxiety caused by poor mental health may have become more apparent.

However, there are several limitations to the current study. First, this study was a cross-sectional study. Therefore, this study pointed out new findings by examining the causal relationships assumed as a model, requiring examinations by longitudinal studies in future research. In particular, as mentioned above, the relationship between mental health states and radiation risk anxiety may have changed due to measures taken immediately after the accident. For example, Fukushima Prefecture has conducted annual surveys on mental health and risk perception of radiation for monitoring the health states of residents. Such databases will become necessary for examining changes in causal relationships over time.

Moreover, this study examined the benefits of resilience in the Fukushima Daiichi nuclear power plant accident, focusing on the factor in the evacuation-directed and non-directed areas. It is because whether people live in the evacuation-directed or non-directed areas make a significant distinction in terms of various environmental factors, including information, economic situation, and living environment, in the context of the Fukushima Daiichi nuclear power plant accident. Although this study did not add individually these factors to avoid an overfitted model, these factors perhaps contribute to an understanding of the resilience effect that this study confirmed. Future research should examine the relationships between these factors and the resilience effects.

Finally, this study focused on radiation risk anxiety and discrimination anxiety as representative psychosocial problems related to the nuclear accident. However, there are many other psychosocial problems in the context of the Fukushima Daiichi accident, such as recalling the experience of the disaster through the news, conflicts among family members and friends, loss of one's hometown, and reluctance to buy food due to rumors. The additional effects of resilience on these issues should be discussed in the future. Furthermore, there are various resilience factors other than the action-oriented approach. Acceptance by others as an evacuee which Takebayashi et al. (9) reported as one of the resilience factors may reduce discrimination anxiety following a disaster. In the future, it will be important to examine the additional effects of various resilience factors to expand the scope of support further.

## Conclusion

This study reported a survey conducted on Fukushima residents 10 years after the nuclear accident. The current study found that the benefits of resilience include not only the recovery of mental health states but also the impact on other psychosocial problems related to a nuclear accident. This finding emphasizes the importance of resilience in providing care for the affected people following a disaster for a decade. In particular, the current study also shows that the action-oriented approach, a resilience factor, has an additional effect on radiation risk anxiety and mental health states. Therefore, the affected people in nuclear accidents can be encouraged to enjoy leisure activities and communicate others actively. If they can also accept life after the accident, they may achieve their successful resilience even after about 10 years. Also, care providers and organizations should help acceptance of the post-disaster context and promote opportunities to participate in various leisure and social activities in the relocated community for care after a nuclear accident.

## Data Availability Statement

The raw data supporting the conclusions of this article will be made available by the authors, without undue reservation.

## Ethics Statement

The studies involving human participants were reviewed and approved by the Ethics Committee of Fukushima Medical University. The participants provided their written informed consent to participate in this study.

## Author Contributions

MMa, SY, and HO conceived and designed this study. SY and HO acquired funding. MMa, CN, YT, HS, SY, and HO designed the questionnaire and conducted the survey. TK conceptualized the study, analyzed the data in the study, and wrote a draft of the manuscript. All authors contributed to the revision of the manuscript and critical discussions and have read and agreed to the published version of the manuscript.

## Funding

This study was funded by JSPS KAKENHI Grant Numbers JP19K10647 and JP18H03051.

## Conflict of Interest

The authors declare that the research was conducted in the absence of any commercial or financial relationships that could be construed as a potential conflict of interest.

## Publisher's Note

All claims expressed in this article are solely those of the authors and do not necessarily represent those of their affiliated organizations, or those of the publisher, the editors and the reviewers. Any product that may be evaluated in this article, or claim that may be made by its manufacturer, is not guaranteed or endorsed by the publisher.
